# Weights of gaur (*Bos gaurus*) and banteng (*Bos javanicus*) killed by tigers in Thailand

**DOI:** 10.1002/ece3.6268

**Published:** 2020-04-16

**Authors:** Supawat Khaewphakdee, Achara Simcharoen, Somphot Duangchantrasiri, Vijak Chimchome, Saksit Simcharoen, James L. D. Smith

**Affiliations:** ^1^ Department of Forest Biology Faculty of Forestry Kasetsart University Bangkok Thailand; ^2^ Department of National Parks Wildlife and Plant Conservation Nakhonsawan Thailand; ^3^ Department of Fisheries, Wildlife, and Conservation Biology University of Minnesota Minneapolis MN USA

**Keywords:** large ungulate, prey biomass, tiger, WEFCOM

## Abstract

The primary prey of tigers across much of South‐East Asia has been depleted, reducing the ability of already limited habitat to support tigers. To better understand the extent to which two of the largest prey species, gaur (*Bos gaurus*) and banteng (*Bos javanicus*), contribute to the tiger's diet, we estimated the average size of these species killed by tigers. This information is needed to more accurately calculate biomass of these species in the tiger's diet and to devise strategies to increase tiger carrying capacity where habitat is fragmented and limited in west‐central Thailand. We used temporally clumped locations of 24 satellite radio‐collared tigers to identify their kill sites and obtained mandibles from 82 gaur and 79 banteng. Kills were aged by teeth eruption sequence, sectioning the M1 molar and counting cementum annuli. Of all gaur killed, 45.2% were adults; of all banteng killed, 55.7% were adults. The average weight of banteng killed was 423.9 kg, which was similar to the 397.9 kg average weight for gaur. The mean weight of both prey species is 3.5–4.5 times greater than the predicted 1:1 preferred prey to predator ratio. In the absence of medium‐sized prey, killing these larger animals may be especially critical for female tigers provisioning nearly independent young when male offspring are already larger than the mother. This is the first study to present data on the average weights of gaur and banteng killed in South‐East Asia, and these results suggest that these are key prey species to target in tiger prey recovery efforts.

## Graphical abstract

This is the first study to present data on the average weights of gaur and banteng killed in South‐East Asia, and these data suggest that gaur and banteng are critical prey species to target to facilitate tiger prey recovery efforts.

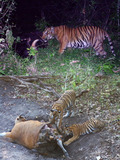

## 1 INTRODUCTION

Across the tiger's range, habitat loss, degradation, and fragmentation have resulted in reduced habitat units that support smaller more isolated tiger populations. Conservation efforts are focused on reversing these trends by enlarging the land base that supports individual tiger populations through community forest and buffer zone programs, the addition of protected areas and by increasing connectivity between or among populations. However, most tiger populations are isolated from each other (Ranganathan, Chan, Karanth, & Smith, 2008), and thus, conservation of this endangered felid is largely an independent outcome of management efforts to conserve each separate population. Often the land is not available to increase the extent of habitat or improve connectivity. In this situation, the practical alternative is to improve habitat quality. This action consists of either restoring degraded habitat so that it supports a higher biomass of prey or through a reduction in poaching of both prey and tigers. The goal of all these activities is to increase the carrying capacity of the habitat so that the size, and thus the viability, of a population is augmented in a spatially limited land base.

As a starting point for improving tiger carrying capacity, to paraphrase Chakrabarti et al. (2016), we need to know “what and how much” a tiger eats. This knowledge, combined with prey and habitat assessments, provides managers with the information to estimate current and potential future tiger carrying capacity of a specific reserve or landscape. Past studies of what and how much a tiger eats analyzed tiger diet by collecting scats to determine prey richness and to identify the tiger's primary prey. Diet studies typically estimate the biomass contribution of each prey species in a tiger's diet by analyzing scats to determine the biomass a single scat of each species represents.

McNab (1980) investigated the nonlinear relationship between surface area and biomass of mammals in terms of energetic needs of a species. This same surface area to mass relationship determines the biomass of prey that a single scat represents. Floyd, Mech, and Jordan (1978) used feeding trials to estimate the biomass of a prey species a single wolf scat represented. A few years later, Ackerman, Lindzey, and Hemker (1984) modified this approach to develop a regression equation for mountain lions (*Puma concolor*). Their model has been used widely to estimate the biomass in the diet of many large felid species including most tiger diet studies prior to 2016. More recently, Chakrabarti et al. (2016) revised Ackerman et al.’s (1984) analysis to take into account satiation, prey digestibility, and carcass use by other carnivores that scavenge kills of primarily large prey. This revision suggests that biomass in a scat reaches an asymptote where the biomass per scat does not increase with increasing prey size. The model from Chakrabarti et al. (2016) is based on the average ratio of prey weight to carnivore weight. Our study considers the impact of average prey weights of gaur and banteng, two of the largest tiger prey, on the diet of tigers in both the models (Ackerman et al., 1984; Chakrabarti et al., 2016).

**FIGURE 1:**
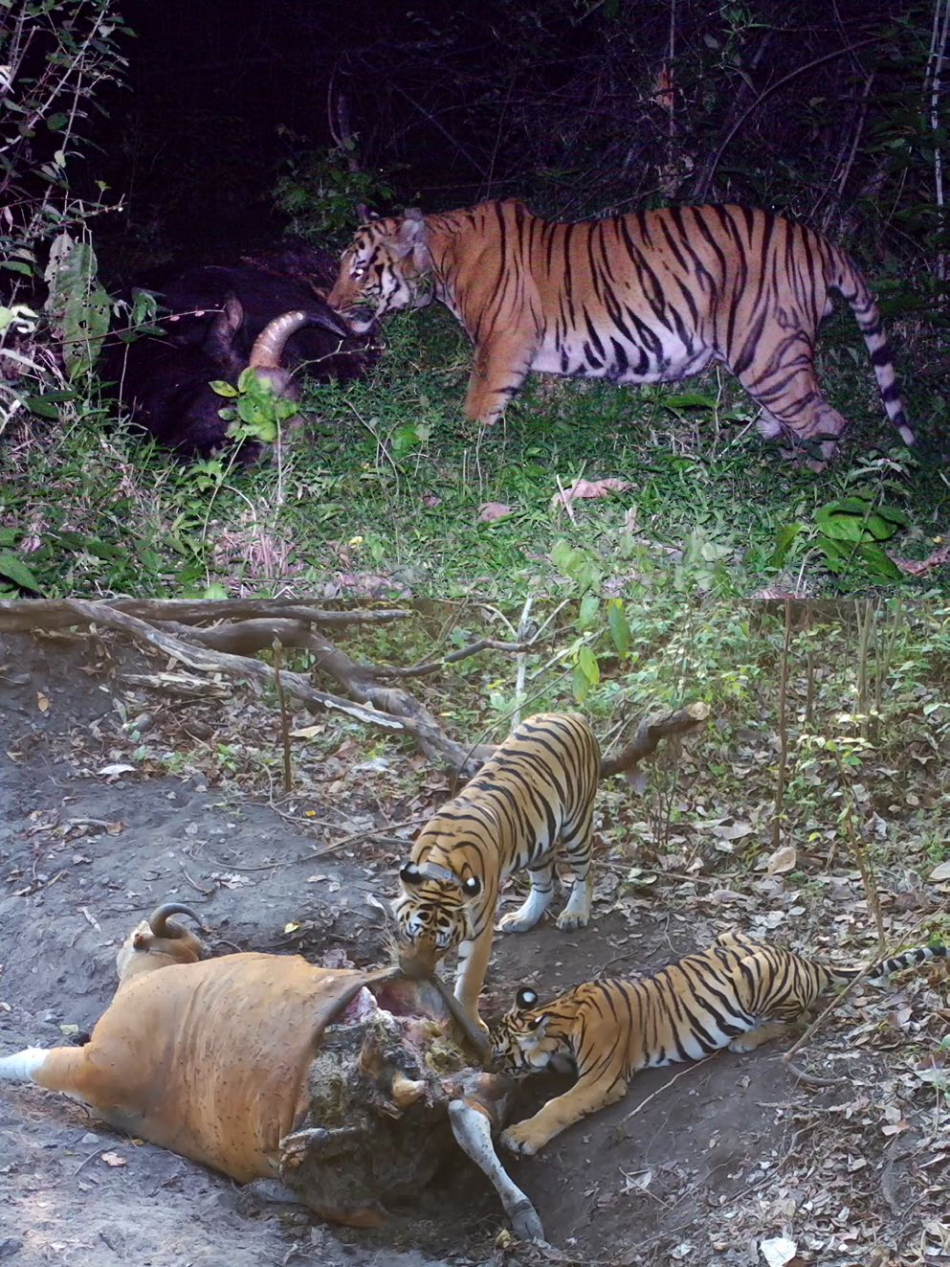
Two of the largest prey of tiger in Huai Kha Khaeng (a) Male tiger feeding on gaur (*Bos gaurus).* (b) Female tiger and her cubs feeding on banteng (*B. javanicus*)

Both models rely on estimating the average weight of each species consumed by tigers. Karanth and Sunquist (1995) pioneered rigorous estimates of the average weight of the tiger's main prey. They determined the percentage of age and sex classes killed by tigers and used published weights of each age class, to calculate the mean weight of each of the tiger's main prey species. These weights have subsequently been used in most tiger diet studies. In South‐East Asia, a new and accurate estimate of average prey weights, especially of large prey, may be especially important for estimating prey biomass in a tiger's diet because the complement of large‐ and medium‐sized prey classes (Andheria, Karanth, & Kumar, 2007) differs from South Asia. Of note, three medium‐sized deer (*Axis porcinis, Panolia eldi, and Rucervus shcomburki*) have been extirpated in South‐East Asia (IUCN, 2019) resulting in a large prey size gap between wild boar (*Sus scrofa*) and other <38 kg prey and the next larger prey, the sambar (*Rusa unicolor*), whose average weight is estimated to be 212 kg (Karanth & Sunquist, 1995). In South‐East Asia, two of the three largest tiger prey are gaur (*Bos gaurus*) and banteng (*Bos javanicus*) (Figure 1). Diet information, specific to South‐East Asia is needed to improve understanding of how to increase tiger carrying capacity in this region where habitat is fragmented and limited.

This study estimated mean weights of gaur and banteng killed by tigers. These averages will help elucidate the role of these two of the largest prey species in the regional diet of tigers. Using the same approach as Karanth and Sunquist (1995), we estimated the proportion of sex and age class of gaur and banteng at tiger kill sites and obtained published weights of each age and sex class. The proportion of these size classes also provides insight into tiger predation strategies. Carbone, Pettorelli, and Stephens (2011) generalized that the prey to predator biomass ratio has a stronger impact on larger carnivores than it does on smaller carnivores. Hayward et al. (2006), Hayward, O’Brien, and Kerley (2007) refined this generality to predict the optimum prey sizes for several large carnivores; for tigers, the ratio was reported to be 1:1 (Hayward, Jedrzejewski, & Jedrzewska, 2012). These generalizations provide insight into evolutionary dynamics of these species, but the additional challenge for conservation biologists and managers is to understand the factors that result in a deviation from these generalities that are important to conservation. For example, in South‐East Asia, tigers depend more on larger prey than in South Asia due to the extirpation of three medium‐sized species of deer (Simcharoen, Simcharoen, Duangchantrasiri, Bump, & Smith, 2018).

Using dental annuli and horn characteristics to classify age and sex of gaur and banteng, combined with published weights for these classes (Ahrestani, 2018; Hoogerwerf, 1970), enabled us to calculate the average weights of gaur and banteng killed by tigers. These weights are key to estimating the biomass of these species in the diet of tigers. Because adult male gaur are 1.3 times larger than adult male banteng, and males of both species are >3.5 times larger than Hayward et al. (2012) optimum 1:1 predator to prey ratio, we hypothesize that these species, and especially the large size classes, are approaching the maximum size limit of tiger prey. Support for this hypothesis would be (a) male tigers kill a smaller percentage of larger adult male gaur than smaller adult banteng, (b) female tigers, which average two thirds the weight of adult male tigers, kill a smaller percentage of adult males of both species compared with male tigers, and (c) female tigers kill fewer males of both species compared with males. It is important to note that our research does not imply prey preference (Hayward et al., 2012); we simply report size, sex, and age class of gaur and banteng killed by tigers in our study area.

## 2 MATERIALS AND METHODS

### 2.1 Study area

**FIGURE 2:**
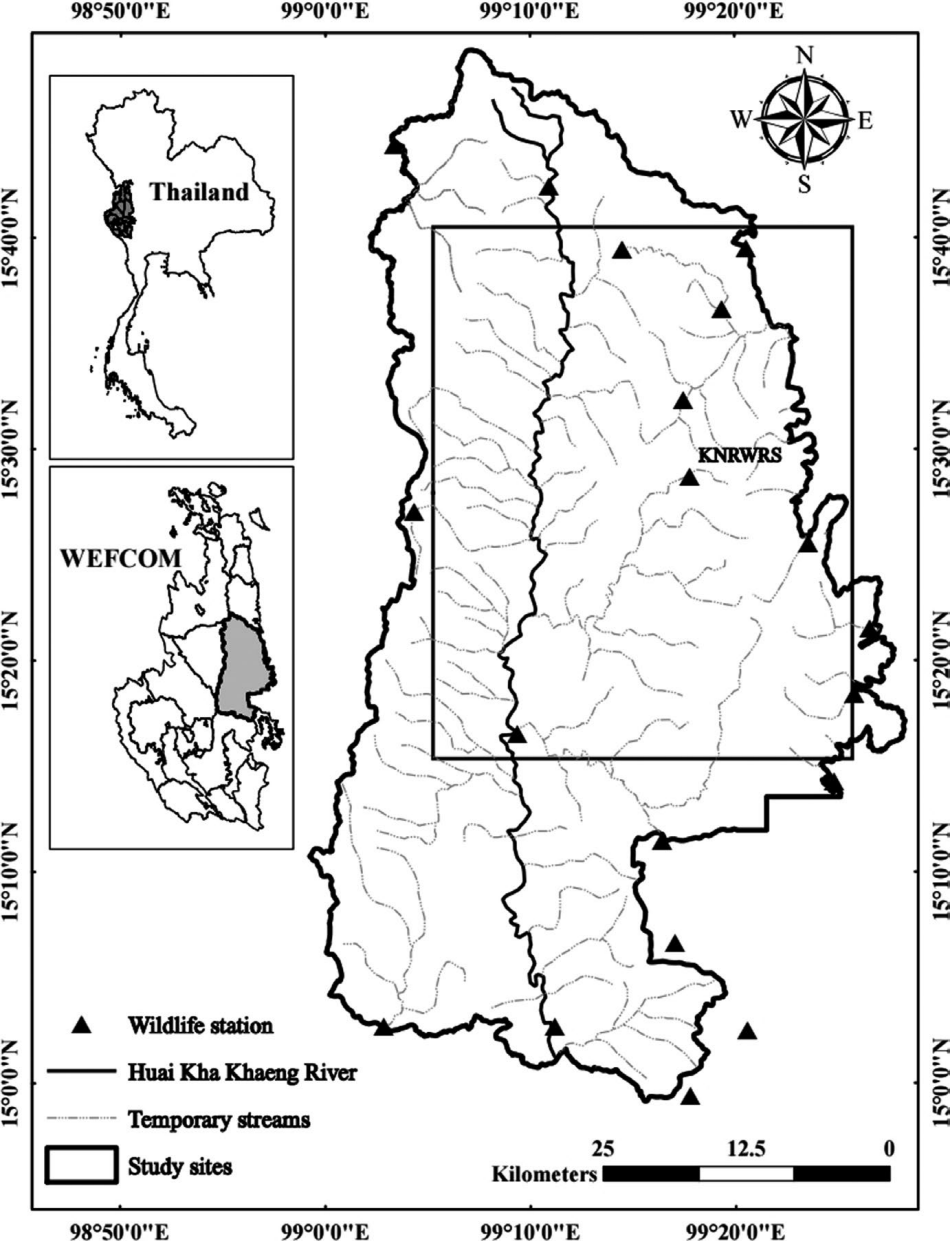
Location of Huai Kha Khaeng Wildlife Sanctuary (HKK) and Khao Nang Rum Wildlife Research Station (KNRWRS) in the Western Forest Complex (WEFCOM), Thailand

The study was conducted in Huai Kha Khaeng Wildlife Sanctuary (HKK) (2,780 km^2^) and has the highest density of tigers in Thailand (1.25–2.01/100 km^2^) (Duangchantrasiri et al., 2016). HKK is also the core sanctuary in a complex of 17 protected areas that form the 18,727 km^2^ Western Forest Complex (WEFCOM) (Figure 2). This complex supports the largest tiger population in Thailand (DNP, 2010) and perhaps the 4th largest globally (Kenney, Allendorf, McDougal, & Smith, 2014). HKK combined with adjacent reserves, Thung Yai Naresuan East Wildlife Sanctuary and Thung Yai Naresuan West Wildlife Sanctuary, is considered a source for re‐establishing tigers throughout the region. Much of the rest of WEFCOM is subject to prey depletion from poaching, and therefore, it is estimated that tigers occur in only 37% of WEFCOM (Duangchatrasiri et al., 2019). HKK is composed of four main vegetation types: mixed deciduous forest (48.3%), dry evergreen forest (24.7%), hill evergreen forest (13.4%), and dry deciduous dipterocarp forest 6.9% (Trisurat, 2004). Although elevation ranges from 200 to 2,180 m, it generally varies from 600 to 1,000 m. HKK has South‐East Asia's highest diversity of large ungulates which include gaur, banteng, sambar, and water buffalo (*Bubalus bubalis*); together these species compose 90.3% of tiger diet. An additional 12 species constitute the other 9.7% of the diet (Simcharoen et al., 2018). Pakpien et al. (2017) documented 11 of the tiger's 16 known prey species at kill sites. Our study site coincided geographically with these two research locations.

### 2.2 Data collection and analysis

**FIGURE 3:**
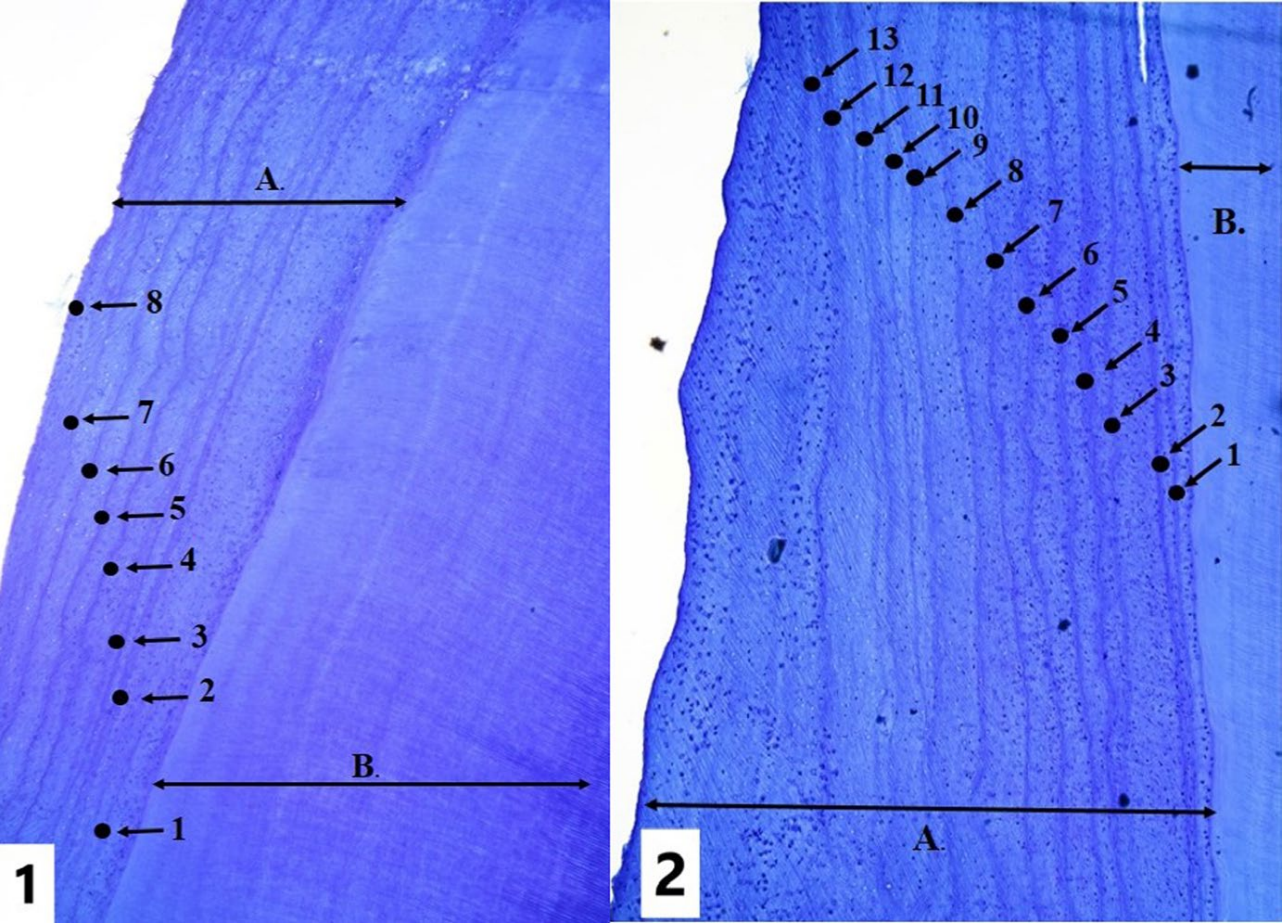
Samples (1) and (2) are from banteng; the left is eight years old (BHKK28); the right is 13 years old (BHKK53). A = cementum, B = dentine, black dots = cementum annuli

At kill sites (15 00′–15 40′ N, 99 00′–99 25′ E), we collected the lower mandible of each gaur or banteng for aging. We determined sex of adult animals based on the configuration of horns (Ahrestani, 2018). The ages of calves and juveniles were determined by teeth eruptions sequence (Dyce, Sack, & Wensing, 2009). For adult prey, we extracted the first molar from the mandible, washed the tooth in water, decalcified it in a weak acid solution (HNO3 5%), and finally rinsed it again in water to stop decalcification (Klevezal, 1996; Spinage, 1976). Each molar was then dehydrated in isopropyl alcohol, frozen, and sectioned with a microtome to create 15–20 µm longitudinal cross‐sectional slices that were mounted on a glass slide. Slices were stained with Giemsa blood and labeled; stained sections were subsequently examined at 10x magnification, and cementum annuli were counted (Figure 3). We concluded that the local single rainy season resulted in a single annuli pattern in western Thailand and confirmed this by comparing annuli data to horn patterns (Ahrestani & Prins, 2011). A single cementum annuli in tropical ungulates was first reported by Spinage (1976). We also followed Ahrestani and Prins (2011) in grouping ages into the following classes: calves (0–1 year), juveniles (>1 to 3 years), young adult (>3 to 6 years), and mature adults (>6‐years).

To determine mean prey size killed by tigers, we multiplied the frequency of each age class, or for adults the sex and age class, by the estimated weight of that class. Age class weights were derived from Ahrestani (2018) and Hoogerwerf (1970). Estimated mean sizes of prey species killed by tigers are used by Ackerman et al. (1984) and Chakrabarti et al. (2016) to calculate the biomass represented by a single scat of each prey species. We also compared the mean weight of gaur and banteng killed by tigers. To evaluate the hypothesis that gaur and banteng are approaching the limit of prey size killed by tigers, we conducted 3 chi‐squared tests comparing the number of kills by male and female tigers of adult male gaur and banteng.

## 3 RESULTS

From June 2005 to May 2017, we visited kill sites of 24 radio—collared tigers (9 males and 15 females) and recorded a total of 82 gaur and 79 banteng kills based on carcass or skeletal remains. Of all gaur killed, 15.9% were adult males and 29.3% were adult females; adult male banteng comprised 29.1% of kills and 26.6% of kills were adult females. (Table 1). In contrast, calves composed 39% of gaur kills versus 26.6% of banteng kills. As a consequence, despite the fact gaur males were approximately 1.3 times heavier than male banteng, and gaur females were 1.1 times the weight of female banteng, the average weights of both gaur killed (397.9 kg) was less than the average weight of banteng killed (423.9 kg) (Table 1).

**TABLE 1:** Mean weights of gaur (*Bos gaurus*) and banteng (*B. javanicus*) killed by tigers based on number of kills in different sex and age classes and published weights of those classes from Ahrestani (2018) and Hoogerwerf (1970)

Species	Adult male			Adult female			Young adult			Juvenile			Calf			Mean weight
	>6 years			>6 years			3–6 years			>1 to 3 years			0–1 year			
	*N*	%	wt	*N*	%	wt	*N*	%	wt	*N*	%	wt	*N*	%	wt	
Gaur	13	25.6	900	24	19.5	650	6	7.3	388	7	8.5	200	32	39.0	50	397.9
Banteng	23	32.9	700	21	22.8	600	6	7.6	356	8	10.1	200	21	26.6	50	423.9

Based on the average sex and age class weights, and the number of kills in each class, the average weight of adult gaur kills was 737.8 kg and they composed 83.7% of the biomass of gaur killed by tigers. Similarly, the mean adult banteng killed weighted 652.2 kg and adults composed 85.6% of biomass of this species killed by tigers. Adults composed 48.8% of gaur and 79.4% of banteng killed by male tigers; whereas, adult gaur and banteng composed 41.1% and 37.8% of female kills, respectively (Table 2).

**TABLE 2:** Percentage and numbers of sexes and age classes of gaur (*B. gaurus and B. Javanicus*) killed by male and female tigers

Tiger sex	species	Male >6 years	Female >6 years	Yg. Ad >3 to 6 years	Juv. >1 to 3 years	Calves 0–1 year
Male	Gaur	20.9% (9)	27.9% (12)	11.6% (5)	9.3% (4)	30.2% (13)
Female		10.3% (4)	30.8% (12)’	2.6% (1)	7.7% (3)	48.7% (19)
Male	Banteng	47.1% (16)	32.4% (11)	8.8% (3)	2.9% (1)	8.8% (3)
Female		15.6% (7)	22.2% (10)	6.7% (3)	15.6% (7)	40.0% (18)

None of the prediction that we suggested would be support for the hypothesis that gaur and banteng are approaching the size limit of tiger prey were significant. Prediction 1: Male tigers killed fewer adult gaur (*n* = 21) compared with adult banteng (*n* = 27), but the difference was not significant (*χ* ^2^ = 0.75 (1), *p* = .386). Prediction 2: Females also killed fewer adult male bovids (*n* = 11) than adult female bovids (*n* = 21), but the difference was not significant (*χ* ^2^ = 2.133 (1), *p* = .063). Prediction 3: Females did not kill significantly fewer adult gaur and banteng (*n* = 33) than male tigers did (*n* = 48) (*χ* ^2^ = 3.13 (1), *p* < .090). Given that our three predictions were not supported by significant results, our hypothesis that gaur and banteng are near the upper size limit of tiger prey is not confirmed.

## 4 DISCUSSION

An important contribution of this study is the re‐evaluation of the role of bovids in the diet of tigers. Most tiger populations are small and near a critical size threshold where viability is threatened (Kenney et al., 2014). Furthermore, the majority of these small populations currently lack suitable habitat outside of reserves to expand the land base needed to support larger, more viable populations (Ranganathan et al., 2008). In this situation, the primary option for managers who seek to increase population size is to improve habitat carrying capacity. To accomplish this, it is critical to know which species tigers eat and the importance of each species in the diet of tigers. This information comes primarily from previously published diet studies. For example, Simcharoen et al. (2018) reported that gaur and banteng, two of the largest prey that tigers consume, compose 46%–59% of the tiger's diet. However, in that study, the authors used Karanth and Sunquist’s (1995) average gaur weight of 287 kg for both gaur and banteng. Our study estimated the average weight of these species in Thailand following a similar approach to Karanth and Sunquist (1995). A reassessment of mean weight of these species killed by tigers was needed because Sunquist and Karanth's estimate was the only previous, rigorous estimate published. Furthermore, the biomass of large bovids in the tiger's diet is higher in South‐East Asia compared with South Asia because a medium‐sized prey, common in the tiger's diet in South Asia (e.g., *Axis axis*), is lacking. We found the average size of gaur killed by tigers in South‐East Asia is nearly 1.35 times greater than the average size in South Asia. This difference may be attributed to the fact that adult male gaur represent only 14.6% of gaur kills in South Asia versus 25.5% of kills in South‐East Asia. In contrast in South Asia, 58% of kills were calves versus 39% in South‐East Asia. We do not speculate on these differences as they could reflect a variety of ecological or behavioral differences (e.g., degree of cover, herd sizes).

An important question is how does our revised estimate of mean prey size of these two tiger preys impact tiger diet estimates and potential tiger habitat carrying capacity. Using Ackerman et al. (1984) linear equation, our re‐estimate of the mean weight of gaur and banteng killed by tigers increases the estimated biomass per scat from 12 to approximately 16 kg per scat, thus substantially increasing the biomass estimate of these two species in the tiger diet. However, Chakrabarti et al. (2016) regression of scat biomass weights does not increase the per scat biomass because their equation reaches an asymptote when the prey's weight is approximately equal to the weight of the predator. Chakrabarti and colleagues make a persuasive argument that tigers may reach satiation (“gut fill”) before completely consuming an entire gaur or banteng carcass. They extend their argument with the suggestion: “since handling costs and risk of injuries usually increase with prey size (Hayward & Kerley, 2005), we postulate that tropical felids would tend to gain more by predating on prey roughly equal to or less than their own sizes and not from larger prey.”

Hayward and Kerley (2005) make a cogent argument, but why then do tigers in Thailand kill a major proportion of their prey that weighs 4–4.5 times more than their own average body weight, thus deviating from Hayward et al. (2012) prediction of preferred prey size? The answer may be, they kill what is available. Although female tigers killed fewer adult gaur and banteng (*n* = 33) than male tigers (*n* = 48), they did kill 11 adult male bovids (31% of adult male bovids killed by tiger). Ackerman et al.’s (1984) prey biomass model does not address gut fill, but according to Chakrabarti et al.’s (2016) model, a large portion of the 81 adult gaur and banteng kills are not utilized by tigers. These authors suggest that scavengers play a role in consumption of larger tiger kills. We agree. Tigers often leave carcasses in the heat of the day to seek shade, drink water, and rest, particularly when a kill is made more open environments. Scavengers use these opportunities to feed on tiger carcasses. In contrast, if a female with large cubs kills in dense cover near water, she and her offspring are likely to remain nearby and there is little opportunity for other species to scavenge. However, neither Chakrabarti et al.’s or Ackerman et al.’s research, or previous diet studies, have adequately estimated the role of scavenging or its impact on the biomass of large prey in the tiger's diet. Camera trap photographs have documented many other species at kill sites; for example, we recently recorded three Asian water monitors (*Varanus salvator*) (~1.4 m long) and several wild boars (*Sus scrofa*) scavenging an adult banteng kill between tiger feeding bouts at HKK (unpublished data).

Another unexplored factor in Chakrabarti and colleagues’ model is the mean weight of tigers; they used an average tiger weight estimated by Prater and Barruel (1971). In their model, our re‐estimate of gaur and banteng weights may not impact diet estimates, because gut fill and scavenging limit tiger utilization of large bovids. However, our new weight estimates may be important when taking into account the combined consumption of a female and large cubs feeding on a kill. Female tigers, often with interbirth interval of <22 months (Kerley, Cowling, Boshoff, & Sims‐Catley, 2003; Smith & McDougal, 1991), are rarely without cubs. Furthermore, tigresses continue to be the primary provider when young males are larger than their mother and daughters are nearly her size. With 2–3 offspring, a 136 kg female is providing for the equivalent of 3–4 adults just prior to her litter becoming independent; their combined family weight is 408–544 kg. If Chakrabarti's model were adjusted to reflect the mean weight of a mother and her young, it would clarify the role of large bovids in the diet of tigers. We plan to collaborate with Chakrabarti in the future to incorporate family size and scavenging in diet studies.

## 5 CONSERVATION IMPLICATIONS

Our reassessment of average prey weights of gaur and banteng killed by tigers may impact estimate of the proportion of biomass in the diet of tigers. Tiger diet studies have traditionally relied on the average weights of prey species estimated by Karanth and Sunquist (1995) at a single location in India. Other tiger diet studies have used different average prey weights but provided little supporting methodology for these estimates. Given widespread prey depletion (Karanth & Stith, 1999) coupled with the reduced land base supporting many tiger populations and limited opportunities to increase habitat connectivity, a careful assessment of all aspects of the food base for tigers is needed. Rigorous models for determining the biomass of species in a tiger's diet need to be supported by accurate field data on the average weight of species killed and the proportion of carcasses consumed. Together this information will allow managers to give the same attention to prioritizing prey species for restoration that this topic has received in North America, Europe, and Australia (Di Marco et al., 2017). Our study suggests that gaur and banteng are important target species for conservation efforts that restore the tiger's prey base in South‐East Asia. Furthermore, Duangchatrasiri et al. (2019) and Jornburom (2016) have shown that the restoration of these bovids can have a major impact on the distribution of tigers in WEFCOM.

## CONFLICT OF INTEREST

There is no conflict of interest.

## AUTHOR CONTRIBUTION

**Supawat Khaewphakdee:** Data curation (equal); Formal analysis (equal); Methodology (equal); Writing‐original draft (equal); Writing‐review & editing (equal). **Achara Simcharoen:** Conceptualization (equal); Data curation (equal); Formal analysis (equal); Funding acquisition (equal); Investigation (equal); Methodology (equal); Project administration (equal); Resources (equal); Supervision (equal); Writing‐original draft (equal); Writing‐review & editing (equal). **Somphot Duangchantrasiri:** Conceptualization (supporting); Data curation (equal); Formal analysis (supporting); Methodology (supporting); Project administration (equal). **Vijak Chimchome:** Methodology (supporting); Supervision (supporting); Writing‐original draft (supporting); Writing‐review & editing (supporting). **Saksit Simcharoen:** Conceptualization (equal); Data curation (equal); Formal analysis (supporting); Methodology (supporting); Project administration (supporting). **James L. D. Smith:** Conceptualization (supporting); Formal analysis (equal); Funding acquisition (equal); Methodology (supporting); Supervision (equal); Writing‐original draft (equal); Writing‐review & editing (equal). 

## Data Availability

Age of gaur and banteng as prey of tiger: Data available from Dryad Digital Repository https://doi.org/10.5061/dryad.mpg4f4qwl.

## References

[ece36268-bib-0001] Ackerman, B. B. , Lindzey, F. G. , & Hemker, T. P. (1984). Cougar food habits in southern Utah. Journal of Wildlife Management, 48(1), 147–155. 10.2307/3808462

[ece36268-bib-0002] Ahrestani, F. S. (2018). *Bos frontalis* and *Bos gaurus* (Artiodactyla: Bovidae). Mammalian Species, 50(959), 34–50. 10.1093/mspecies/sey004

[ece36268-bib-0003] Ahrestani, F. S. , & Prins, H. H. T. (2011). Age and sex determination of gaur *Bos Gaurus* (Bovidae). Mammalia, 75, 151–155. 10.1515/MAMM.2010.078

[ece36268-bib-0004] Andheria, A. P. , Karanth, K. U. , & Kumar, N. S. (2007). Diet and prey profiles of three sympatric large carnivores in Bandipur Tiger Reserve, India. Journal of Zoology, 273(2), 169–175. 10.1111/j.1469-7998.2007.00310.x

[ece36268-bib-0005] Carbone, C. , Pettorelli, N. , & Stephens, P. A. (2011). The bigger they come, the harder they fall: Body size and prey abundance influence predator–prey ratios. Biology Letters, 7, 312–315. 10.1098/rsbl.2010.0996 21106569 PMC3061189

[ece36268-bib-0006] Chakrabarti, S. , Jhala, Y. V. , Dutta, S. , Qureshi, Q. , Kadivar, R. F. , & Rana, V. J. (2016). Adding constraints to predation through allometric relation of scats to consumption. Journal of Animal Ecology, 85, 660–670. 10.1111/1365-2656.12508 26931378

[ece36268-bib-0007] Di Marco, M. , Chapman, S. , Althor, G. , Kearney, S. , Besancon, C. , Butt, N. , … Watson, J. E. M. (2017). Changing trends and persisting biases in three decades of conservation science. Global Ecology and Conservation, 10, 32–42. 10.1016/j.gecco.2017.01.008

[ece36268-bib-0008] DNP . (2010). Thailand tiger action plan 2010–2022. Bangkok, Thailand: Department of National Parks, Wildlife and plant conservation.

[ece36268-bib-0009] Duangchantrasiri, S. , Umponjan, M. , Simcharoen, S. , Pattanavibool, A. , Chaiwattana, S. , Maneerat, S. , … Karanth, K. U. (2016). Dynamics of a low‐density tiger population in Southeast Asia in the context of improved law enforcement. Conservation Biology, 30, 639–648. 10.1111/cobi.12655 27153529

[ece36268-bib-0010] Duangchatrasiri, S. , Jornburom, P. , Jinamoy, S. , Pattanvibool, A. , Hines, J. E. , Arnold, T. W. , … Smith, J. L. D. (2019). Impact of prey occupancy and other ecological and anthropogenic factors on tiger distribution in Thailand's western forest complex. Ecology and Evolution, 9, 2449–2458. 10.1002/ece3.4845 30891192 PMC6405490

[ece36268-bib-0011] Dyce, K. M. , Sack, W. O. , & Wensing, C. J. G. (2009). Textbook of veterinary anatomy (4th ed.). St. Louis, Missouri: P. Rudolph.

[ece36268-bib-0012] Floyd, T. J. , Mech, L. D. , & Jordan, P. A. (1978). Relating wolf scat content to prey consumed. Journal Wildlife Management, 42(3), 528–532. 10.2307/3800814

[ece36268-bib-0014] Hayward, M. W. , Henschel, P. , O’Brien, J. , Hofmeyr, M. , Balme, G. , & Kerley, G. I. H. (2006). Prey preferences of the leopard (*Panthera pardus*). Journal of Zoology, 270, 298–313. 10.1111/j.1469-7998.2006.00139.x

[ece36268-bib-0015] Hayward, M. W. , Jedrzejewski, W. , & Jedrzewska, B. (2012). Prey preferences of the tiger *Panthera tigris* . Journal of Zoology, 286, 221–231. 10.1111/j.1469-7998.2011.00871.x

[ece36268-bib-0016] Hayward, M. W. , & Kerley, G. I. H. (2005). Prey preferences of the lion (*Panthera leo*). Journal Zoological Society of London, 267, 309–322. 10.1017/S0952836905007508

[ece36268-bib-0017] Hayward, M. W. , O’Brien, J. , & Kerley, G. I. H. (2007). Carrying capacity of large African predators: Predictions and tests. Biological Conservation, 139, 219–229. 10.1016/j.biocon.2007.06.018

[ece36268-bib-0018] Hoogerwerf, A. (1970). Udjung Kulon: The land of the last javan rhinoceros. Leiden, Netherlands: E. J. Brill.

[ece36268-bib-0019] IUCN . (2019). The IUCN Red List of Threatened Species. Version 2019–3. Retrieved from http://www.iucnredlist.org

[ece36268-bib-0020] Jornburom, P. (2016). The distribution of elephants, tigers and tiger prey in Thailand’s western forest complex. St. Paul, Minnesota: University of Minnesota. PhD dissertation.

[ece36268-bib-0021] Karanth, K. U. , & Stith, B. M. (1999). Prey depletion as a critical determination of tiger population viability In Seidensticker J., Christie S., & Jackson P. (Eds.), Riding the tiger: Conservation in human dominated landscapes (pp. 100–113). Cambridge, U.K.: Cambridge University Press.

[ece36268-bib-0022] Karanth, K. U. , & Sunquist, M. E. (1995). Prey selection by tiger, leopard and dhole in tropical forests. Journal of Animal Ecology, 64(4), 439–450. 10.2307/5647

[ece36268-bib-0023] Kenney, J. , Allendorf, F. W. , McDougal, C. , & Smith, J. L. D. (2014). How much gene flow is needed to avoid inbreeding depression in wild tiger populations? Proceedings of the Royal Society B: Biological Sciences, 281, 20133337 10.1098/rspb.2013.3337 PMC4100497 24990671

[ece36268-bib-0024] Kerley, G. L. H. , Cowling, R. L. M. , Boshoff, A. F. , & Sims‐Catley, R. (2003). Options for the conservation of large and medium‐sized mammals in the Cape Floristic Region hotspot, South Africa. Biological Conservation, 112, 169–190. 10.1016/s0006-3207(02)00426-3

[ece36268-bib-0025] Klevezal, G. A. (1996). Recording structures of mammals: Determination of age and econstruction of life history. Rotterdam, Netherlands: A. A. Balkema.

[ece36268-bib-0026] McNab, B. K. (1980). Food habits, energetics, and the population biology of mammals. American Naturalist, 116(1), 106–124. 10.1086/283614

[ece36268-bib-0027] Pakpien, S. , Simcharoen, A. , Duangchantrasiri, S. , Chimchome, V. , Pongpattannurak, N. , & Smith, J. L. D. (2017). Ecological Covariates at Kill Sites Influence Tiger (*Panthera tigris*) Hunting Success in Huai Kha Khaeng Wildlife Sanctuary, Thailand. Tropical Conservation Science, 10, 1–7. 10.1177/1940082917719000

[ece36268-bib-0028] Prater, S. H. , & Barruel, P. (1971). The book of indian animals (3rd ed.). Bombay, India: Natural History Society.

[ece36268-bib-0029] Ranganathan, J. , Chan, K. M. A. , Karanth, K. U. , & Smith, J. L. D. (2008). Where can tigers persist in the future? A landscape‐scale, density‐based population model for the Indian subcontinent. Biological Conservation, 141(1), 67–77. 10.1016/j.biocon.2007.09.003

[ece36268-bib-0030] Simcharoen, A. , Simcharoen, S. , Duangchantrasiri, S. , Bump, J. , & Smith, J. L. D. (2018). Tiger and leopard diets in western Thailand: Evidence for overlap and potential consequences. Food Webs, 15, e00085 10.1016/j.fooweb.2018.e00085

[ece36268-bib-0031] Smith, J. L. D. , & McDougal, C. (1991). The contribution of variance in lifetime reproduction to effective population size in tigers. Conservation Biology, 5, 484–490. 10.1111/j.1523-1739.1991.tb00355.x

[ece36268-bib-0032] Spinage, C. A. (1976). Incremental cementum lines in the teeth of tropical African mammals. Journal of Zoology Society of London, 178, 117–131. 10.1111/j.1469-7998.1976.tb02267.x

[ece36268-bib-0033] Trisurat, Y. (2004). GIS database and its applications for ecosystem management. Bangkok, Thailand: The Western Forest Complex Ecosystem Management Project (WWFCOM), National Park, Wildlife and Plant Conservation Department.

